# Impaired Morris water task retention following T21 light dark cycle exposure is not due to reduced hippocampal c-FOS expression

**DOI:** 10.3389/fnbeh.2022.1025388

**Published:** 2022-10-12

**Authors:** Scott H. Deibel, S. Higdon, T. T. S. Cassell, M. L. House-Denine, E. Giberson, I. C. Webb, C. M. Thorpe

**Affiliations:** ^1^Department of Psychology, University of New Brunswick, Fredericton, NB, Canada; ^2^Department of Psychology, Memorial University of Newfoundland, St. John’s, NL, Canada

**Keywords:** circadian rhythms, rats, Morris water maze (MWM), memory impairment, c-FOS, T21 light dark cycle

## Abstract

Circadian rhythms influence virtually all aspects of physiology and behavior. This is problematic when circadian rhythms no longer reliably predict time. Circadian rhythm disruption can impair memory, yet we don’t know how this fully works at the systems and molecular level. When trying to determine the root of a memory impairment, assessing neuronal activation with c-FOS is useful. This has yet to be assessed in the hippocampi of circadian rhythm disrupted rats in a hippocampal gold standard task. Rats were trained on the Morris water task (MWT), then received 6 days of a 21-h day (T21), 13 days of a normal light dark cycle, probe trial, and tissue extraction an hour later. Despite having impaired memory in the probe trial, compared to controls there were no differences in c-FOS expression in hippocampal sub regions: CA1; CA3; Dentate gyrus. These data confirm others in hamsters demonstrating that arrhythmicity which produces an impairment in spontaneous alternation does not affect c-FOS in the dentate gyrus. The current study indicates that the memory impairment induced by a lighting manipulation is likely not due to attenuated neuronal activation. Determining how the master clock in the brain communicates with the hippocampus is needed to untangle the relationship between circadian rhythms and memory.

## Introduction

Since observation of diurnal variations in both human, and rodent memory, circadian rhythms and memory have received much attention (Eckel-Mahan and Storm, 2009; Smarr et al., 2014; Krishnan and Lyons, 2015; Rawashdeh et al., 2018; Snider et al., 2018; Hartsock et al., 2020; Lehr et al., 2021). A common question is whether manipulating circadian rhythms impairs memory. In the 1970s, and 80s several reports found that phase advances and delays of the light dark cycle impaired active and passive avoidance memory in rats (Davies et al., 1974; Tapp and Holloway, 1981; Fekete et al., 1985, 1986; Stone et al., 1992). With its involvement in both episodic memory and Alzheimer’s disease, some wondered whether the hippocampus would be similarly affected. Devan et al. (2001) found that changes in the light dark cycle affected hippocampal dependent memory in rodents. They trained rats on the place version of the Morris water task (MWT) during 6 days of a 21 h daylength (henceforth referred to as T21), which is outside the range of entrainment for the rat. While MWT acquisition was unaffected, retention was impaired 17 days later, despite having had a normal 24 h day-length since the end of training. This finding has been replicated (Devan et al., 2001; Zelinski et al., 2014), and similar designs from other labs (Gibson et al., 2010; Loh et al., 2010; Iggena, 2017; Horsey et al., 2020; Lewis et al., 2020; Haraguchi et al., 2021) also produce memory impairments.

T21 light dark cycles are outside the range of entrainment for rats, meaning that they are oscillating according to their endogenous period (Stephan, 1983; Campuzano, 1998; Craig and McDonald, 2008; McDonald et al., 2013; Zelinski et al., 2014; Deibel et al., 2019). During this type of schedule animals are eating, sleeping, and are active at the same circadian time, but a different zeitgeber time each day. Thus, there is a phase mismatch between the rat’s circadian system and that of the T21 (Deibel et al., 2020).

Much research has been devoted to understanding why memory might be impaired from different types of circadian rhythm disruption. Molecules involved in synaptic plasticity oscillate in the hippocampus and are influenced by clock genes (Rawashdeh et al., 2018; Snider et al., 2018; Hartsock et al., 2020; Lehr et al., 2021). We know that hippocampal clock gene expression is essential for memory retention (Shimizu et al., 2016; Kwapis et al., 2018; Hasegawa et al., 2019; Hartsock et al., 2020). Yet we do not know exactly how hippocampal clock genes are modulated. We have ruled out some possibilities such as an increased stress response (Deibel et al., 2014) and sleep disruption (Deibel et al., 2020). Circadian rhythm disruption induced changes in neuromodulators such as corticosterone, melatonin, dopamine, gamma-aminobutyric have been discussed as likely candidates (Ruby et al., 2008; Cain et al., 2017; Iggena, 2017). Upregulating some of these neuromodulators during conditions of circadian rhythm disruption can rescue memory (Ruby et al., 2008, 2013; Iggena, 2017). But it is unclear if the effect is acting on the hippocampus directly, or indirectly *via* other forces such as the suprachiasmatic nucleus (SCN) (Iggena, 2017).

By understanding more about the nature of the memory impairment induced by a T21, we might be able to determine better how circadian rhythms interact with memory. c-FOS is an immediate early proto-oncogene that is indicative of neuronal activity (Sagar et al., 1988; Hughes and Dragunow, 1995). Quantifying c-FOS expression in the hippocampus is a good step for investigating a memory impairment because it will provide information on whether the effect is due to less neurons being recruited during learning or memory retrieval. Training and recall in the MWM increases expression of c-FOS in the dentate gyrus, Cornus Ammon (CA)1, and CA3 regions of the hippocampus, and suppression of c-FOS expression in the hippocampus of rats can produce deficits in memory retrieval on a discrimination task (Grimm et al., 1997; Carter et al., 2015; Barry et al., 2016).

Ruby et al. (2017) partly addressed this question by examining c-FOS in the hippocampi of hamsters that were arrhythmic. While they found no changes in c-FOS expression in arrhythmic vs. entrained hamsters they only looked in the dentate gyrus during baseline conditions instead of during learning or retention (Ruby et al., 2017). This question needs to be assessed during learning and/or retention in more hippocampal subfields, with a task that better assesses hippocampal dependent memory. In the current study, we will use a T21 paradigm that produces memory impairments in the MWT (Zelinski et al., 2014), and then assess c-FOS expression in the dentate gyrus, CA1, and CA3 regions of the hippocampus after the probe trial. Our hypothesis is that the memory impairment induced by a T21 light dark cycle is accompanied by attenuated c-FOS expression in all hippocampal subfields.

## Materials and methods

### Subjects

Sixteen male Long-Evans rats weighing approximately 250 grams at the start of the experiment were obtained from Charles River Laboratories (QC, Canada). Upon arrival, rats were pair-housed in individually ventilated cages (32 cm × 35 cm × 18 cm) for two days. Each cage contained corncob bedding (Netco, New York, NY), Crink-l’Nest (The Anderson, Maumee, Ohio), a wooden block, a Nylabone (Nylabone Products, Neptune, NJ), and a piece of plastic pipe for enrichment. The colony rooms were kept at a temperature of 21°C and a humidity of 35%. The rats were randomly assigned to either the control group (*n* = 8) or the T21 (T21) group (*n* = 8) and were transferred to individual housing. Six rats from each group were housed for the remainder of the experiment in a clear plastic cage (17 cm × 40 cm × 16 cm) attached to a running wheel (36 cm in diameter). The contents of each cage were the same as before, except the plastic pipe was not included. Additionally, all running wheels were connected to a computer programmed to constantly record wheel rotations. The remaining two rats in each condition were singly housed in individually ventilated cages for the remainder of the experiment. Once again, these cages contained the same items as the cages the rats were initially housed in. Rats were housed in temperature-controlled colony rooms and were given *ad libitum* access to standard rat food and water. Rats were maintained on a 12:12 LD cycle (lights on 7:00) prior to the photoperiod shift.

All procedures used in the present experiment were conducted in accordance with the Canadian Council of Animal Care Guidelines and were approved by the Institutional Committee on Animal Care at Memorial University.

### Apparatus

#### Morris water task

This apparatus consisted of a circular metal tank (170 cm in diameter × 60 cm deep) held 28 cm above the floor by a wheeled platform (178 cm × 178 cm). The maze was filled with water to approximately 10 cm below the top of the maze and maintained at a temperature of around 21°C. White non-toxic paint was added to the water to ensure it was opaque. In one quadrant, 30 cm from the side of the tank was an escape platform (11 cm in diameter) which remained in that position for all training trials and was removed for the probe test. The platform was made of white tubing that could be adjusted to vary the height of the platform, was filled with sand, and was located in the North-East quadrant for all training trials. The height of the platform was adjusted so that it remained 2 cm below the water level. The room (357 × 592 cm) had numerous visual cues such as shelving, a table, a window, and posters that were consistent throughout the training and testing.

A camera was mounted in the ceiling above the water maze and was connected to Black Magic software (Black Magic Design, version 1.0) to record all trials.

### Procedure

**FIGURE 1:**
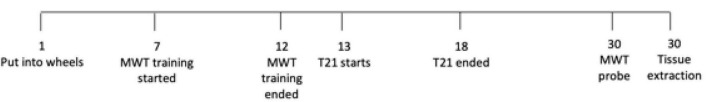
Experimental timeline.

Refer to Figure 1 for an experimental timeline. After being pair housed for 2 days, the rats were then given 7 days to acclimate to being individually housed in wheel cages. Following this 7 day period, rats began acquisition training on the water maze for 6 days. Once the water maze training was completed, control rats were maintained on the same 12:12 LD cycle while T21 rats were subjected to the T21 for 6 days. Rats were then given 13 days to re-entrain to the LD cycle. Rats were not trained or tested in the MWT during the T21 or re-entrainment period. On the day following the 13-day re-entrainment period, a no platform probe test was conducted in the MWT to assess memory retention. All rats were euthanized 1 h following completion of the probe test, and their brains were extracted and analyzed for c-FOS expression using immunohistochemistry (IHC).

#### T21

Throughout the experiment, activity was recorded with running wheels. Rats were housed throughout the initial entrainment and training periods on a 12:12 LD cycle (lights on 7:00 a.m.). Control rats were maintained on this LD cycle for the entirety of the experiment. As in Devan et al. (2001) and Zelinski et al. (2014), a T21 with 12 h of light and 9 h of dark was used (see Table 1). The T21 ended with the lights turning on at 13:00 and off at 1:00, and this schedule was maintained for 13 days until memory retention testing.

**TABLE 1:** T21 schedule.

Day	On	Off	On
1	0400	1,600	
2	0100	1,300	2,200
3		1,000	1,900
4		0700	1,600
5		0400	1,300
6		0100	
6+	1,300	0100	

#### Morris water task

This procedure consisted of two phases, the acquisition training which occurred prior to T21 exposure, and the retention test which occurred following T21 exposure and re-entrainment. Rats were transferred to individually ventilated cages and transported from the colony rooms to the testing room in groups of eight. Throughout the procedure, rats were held in clear conventional cages (45 cm × 25 cm × 21 cm) with metal lids lined with paper towel on the bottom. Training and testing occurred in dim lighting with a radio playing. All training and testing occurred an hour and a half before the lights off time. All trials were analyzed with Ethovision XT (Noldus, version 14.0).

#### Acquisition training

Acquisition training was conducted as described in Zelinski et al. (2014). Each rat received one session daily for six consecutive days at 17:30 [ZT10.5 (zeitgeber time)]. Each session consisted of eight trials, with four release locations around the water maze being used. The release location for the trials were pseudorandomized so that in all sessions the release position changed between rats and every rat was released from each location twice. The escape platform was placed in one quadrant of the maze and was kept in that position for the entirety of the training period. For each trial, rats were carried counter-clockwise around the maze in their cages to a chair at the proper release position and were released into the water as close to the edge as possible with their noses facing the outside of the pool. Rats swam until they either found and climbed upon the escape platform, or 60 s had elapsed, at which point they were guided to the platform by the experimenter. Once rats were on the platform they were left there for about 10 s and then returned to their cage. Between rats the water was agitated to eliminate any odor cues that could be used to locate the platform. Trials were analyzed for the latency to the platform and path length.

#### Retention testing

The day following the 13-day re-entrainment period, a no-platform probe test was performed to assess retention of the platform location following T21 exposure as described in Zelinski et al. (2014). Testing was completed at ZT10.5 (ZT: Zeitgeber time) for both groups, with the control rats tested first at 17:30, followed by the T21 rats at 23:30. For the no-platform probe, the platform was removed from the pool. Each rat received only one trial. Rats were released from one of the two release locations used for acquisition training that were closest to the previous platform location. Release positions were pseudorandomized so that four rats from each condition were released from each position. Rats were carried counter-clockwise in their cages to a chair at the release location and were released into the pool as close to the edge as possible, with their noses facing outward. Each rat swam for 60 s and was then returned to its cage. The probe trials were analyzed for time spent in each quadrant.

#### Tissue collection

One hour following completion of the retention testing, rats were euthanized and tissue was collected as described in Baltazar et al. (2013) with some minor adjustments. An hour after behavior was chosen as the perfusion time because most studies that assess hippocampal c-FOS after behavior, do so 1–1.5 after behavior (Shires and Aggleton, 2008; Lu et al., 2014; Méndez-Couz et al., 2014; Barry et al., 2016; Kalinina et al., 2019; Silva et al., 2019; Tzakis et al., 2020). Due to differences in timing for the retention testing, control rats were euthanized first at 18:30 (ZT11.5), followed by the T21 rats at 00:30 (ZT11.5). Rats were injected intraperitoneally with sodium pentobarbital (150 mg/kg) for anaesthetization. Rats were monitored and deemed deeply anaesthetized once a lack of pedal reflex was demonstrated. Once rats were deeply anaesthetized, they were perfused transcardially with 50 mL saline which was followed by 500 mL of 4% paraformaldehyde in 0.1 M phosphate buffer. The brains were then extracted and post-fixed for 1 h in 4% paraformaldehyde before being transferred to a solution of 20% sucrose with 0.01% sodium azide in 0.1 M phosphate buffer. Brains were stored in this 20% sucrose with 0.01% sodium azide in 0.1 M phosphate buffer solution in a refrigerator at 4°C until they were sliced.

The tissue was sliced with a freezing microtome into four parallel series for each brain. The tissue was sliced into 40 μm coronal sections, starting at the anterior of the brain and continuing posteriorly until the end of the hippocampus. The series were stored in cryoprotectant at –20°C until IHC (Watson et al., 1986).

#### Immunohistochemistry

Sections of each series were stained for c-FOS expression. All procedures were conducted at room temperature. To stain for c-FOS expression, sections were rinsed with 0.1 M phosphate buffer saline (PBS), and then blocked with 1% hydrogen peroxide for 10 min to eliminate any endogenous peroxidase activity. Tissue was then rinsed with 0.1 M PBS and blocked in 0.1 M PBS with 0.4% Triton X-100 (Sigma-Aldrich, St. Louis, MO), and 0.1% bovine serum albumin (PBS+) for 1 h. This blocking was followed by incubation with rabbit anti-c-Fos antibody (Cell Signaling, cat# 2250, 1:1,000) in PBS+ overnight (16 h). The tissue was once again rinsed with PBS and then incubated with biotinylated goat anti-rabbit IgG (GARb, Jackson, 1:500) in PBS+ for 1 h. The tissue was rinsed with 0.1 M PBS before being incubated with avidin-biotin-complex (ABC) elite (Vector, Laboratories) diluted in PBS (1:1,000) for 1 h for signal amplification. The tissue was then rinsed with 0.1 M PBS and incubated with a 3,3′ diaminobendzidine (DAB)-nickel solution containing 0.02% DAB (Sigma-Aldrich), and 0.01% hydrogen peroxide in 0.1 M phosphate buffer for visualization of staining. Finally, the tissue was rinsed with 0.1 M phosphate buffer. The sections were then mounted onto gelatin coated slides, allowed to air dry, dehydrated using a series of graded alcohols, and coverslipped with Permount (Fisher Scientific).

The slides were imaged with a camera (Olympus, BX52) attached to a microscope (Olympus, DP72). Double-blind experimenters selected the dentate gyrus, CA1, and CA3 subregions of the hippocampus, and when possible (tissue was damaged in some cases), within each subregion two sections were sampled from the anterior, middle, and posterior of the dorsal hippocampus (531 out of a maximum of 576 sections counted in total). Thus, for each rat an average of six sections from each subregion were analyzed, for a total of 18 sections per rat. The number of cFOS+ cells in each section was counted bilaterally with Fiji (Schindelin et al., 2012). A threshold was chosen that best labeled the c-FOS+ cells across all the sections. Once determined, the threshold remained constant for all sections and CA1, CA3, and dentate gyrus regions of interest were traced with the drawing function (Paxinos and Watson, 1998). Fiji then counted the number of positive cells in each region of interest. Two experimenters blind to the rat and group identifications scored every section independently.

### Data analyses

SPSS 28 (IBM, Armonk, New York) was used to conduct all the statistical analyses and Prism 9 (GraphPad, La Jolla, CA) was used to make all the figures. Two-tailed statistics with an alpha of.05 were used in all cases. If sphericity was violated, Greenhouse-Geisser corrected values were used. In the case of a significant interaction, simple main effects analyses in the form of Fisher’s LSD comparisons were conducted.

For circadian rhythm analyses, the onset of daily activity and the period of the activity rhythm were calculated with ClockLab Analysis 6 (Actimetrics, Wilmette, IL, USA). With respect to the onset of activity, the program looked for a 5 h period of low activity followed by a 5 h period of high activity. When the program did not accurately identify an onset, it was manually selected. At 6-min intervals, periods between 20 and 28 h were assessed. For wheel analyses, 24 days of assessment were divided into four blocks of 6 days: pre-T21, T21, post T21-1, and post T21-2. Six-day blocks were averaged to produce a mean value per block. Two mixed model ANOVAs with block (× 4 levels) as a repeated measures factor, and light treatment [× 2 levels: 12:12 LD vs. T21 (12:9 LD)] as a between measures factor were used to assesses circadian phase and period, respectively.

For the MWT, the average latency and path length to the platform was calculated for each day of acquisition training. Two mixed model ANOVAs with day (6 levels) as the repeated measures factor and light treatment [2 levels: 12:12 LD vs. T21 (12:9 LD)] were used to assess latency and pathlength. For the 60 s probe trial, the percentage of time spent in the target quadrant and the average percentage of time in the remaining three remaining quadrants were calculated for the first 30 s. A mixed model ANOVA with quadrant (2 levels: target vs. average of remaining three) as the repeated measures factor and light treatment [2 levels: 12:12 LD vs. T21 (12:9 LD)] as the between measures factor.

For every rat, the average number of cFOSs + cells in each hippocampal subregion was calculated to obtain one value per animal per region. These values were then compared between the groups with a single MANOVA containing all hippocampal subregions. A weighted kappa was conducted to assess interrater reliability for cFOS scoring across all sections.

## Results

### Circadian rhythms

**FIGURE 2:**
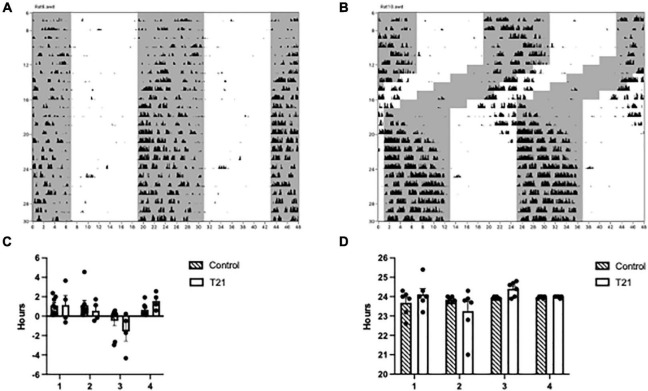
Circadian rhythmicity throughout the experiment. **(A)** Actogram of a control rat throughout the experiment. The gray shaded region represents the portion of the LD cycle when the lights were off. **(B)** Actogram of a T21 rat throughout the experiment. **(C)** Average phase, or time elapsed between lights off and the onset of activity (± SEM) between groups prior to, during and following the T21. A block is equal to 6 days and a negative phase indicates that the activity onset occurred before lights off. **(D)** The average period (±) of circadian rhythms between groups prior to, during, and following the T21.

As depicted in Figure 2 and Supplementary Figures 1, 2, phase changed across the experimental blocks [*F*(3, 30) = 13.758, *p <* 0.0001, η_*p*_^2^ = 0.579], but was not affected by the T21 light dark cycle [*F*(1, 10) = 0.251, *p* = 0.627, η_*p*_^2^ = 0.024]. The experimental block × group interaction, however, was significant [*F*(3, 30) = 3.819, *p* = 0.020, η_*p*_^2^ = 0.276]. Simple main effects analysis indicated that phase was only different in the last 6 days (block 1: *p* = 0.379; block 2: *p* = 0.598; block 3: *p* = 0.266; block 4: *p* = 0.004). Period did not change across the experimental blocks [*F*(1.953, 19.534) = 2.994, *p* = 0.074, η_*p*_^2^ = 0.230], nor was the block × group interaction significant [*F*(1.953, 19.534) = 2.78*3*, *p* = 0.087, η_*p*_^2^ = 0.218]. There was also no difference between the groups [*F*(1, 10) = 0.154, *p* = 0.703, η_*p*_^2^ = 0.015]. No change in period during the 6 days of T21 exposure suggests that the animals were unable to entrain to the T21. A delayed phase in the last 6 days for the T21 rats could indicate that these animals were still not quite adjusted to the new LD cycle.

### Morris water maze

#### Acquisition

**FIGURE 3:**
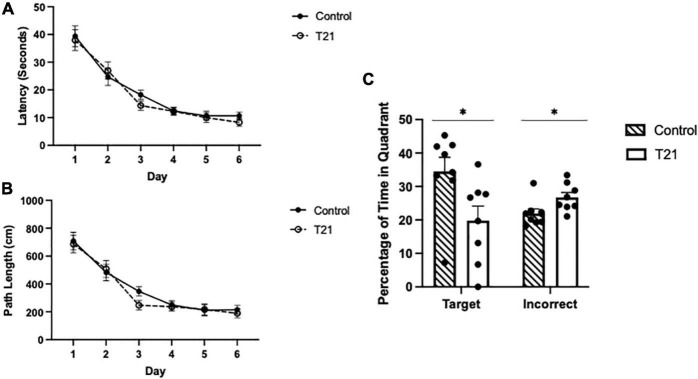
Morris water maze acquisition and retention performance. **(A)** Average latency (± SEM) to reach the platform between groups for each day of acquisition training. **(B)** Average pathlength (± SEM) to reach the platform between groups for each day of acquisition training. **(C)** Average percentage of time spent in the target quadrant (± SEM) and the average percentage of time spent in the other three quadrants (± SEM) during the retention probe between groups. Control rats spent significantly more time in the target quadrant and significantly less time in the other quadrants than T21 rats, *p < 0.05.

As shown in Figure 3, there was a significant main effect of day, [*F*(5, 70) = 60.979, *p* < 0.001, η_*p*_^2^ = 0.813, observed power = 1.00], indicating that latencies decreased as training progressed. Bonferroni corrected *t*-tests confirmed a significant difference between the first and last day of training [*t*(14) = 2.576, *p* < 0.01]. Latencies were not different between the groups [*F*(1, 14) = 0.272, *p* = 0.610, η_*p*_^2^ = 0.019], nor was the day × group [*F*(5, 70) = 0.505, *p* = 0.771, η_*p*_^2^ = 0.035] interaction significant.

The average pathlength to the platform for each day of acquisition training also significantly decreased with training [*F*(5, 70) = 43.794, *p* = 0.000, η_*p*_^2^ = 0.758, observed power = 1.00], but it was not different among the groups [*F*(1, 14) = 0.411, *p* = 0.532, η_*p*_^2^ = 0.029]. Nor was the group × day interaction significant [*F*(5, 70) = 0.511, *p* = 0.767, η_*p*_^2^ = 0.035].

### Retention

As demonstrated in Figure 3, for the probe trial, there was no difference in the percentage of time spent in the target quadrant compared to the average of the remaining three quadrants [*F*(1, 14) = 0.485, *p* = 0.497, η_*p*_^2^ = 0.034]. However, the group × quadrant interaction [*F*(1, 14) = 5.739, *p* = 0.031, η_*p*_^2^ = 0.291, observed power = 0.606], and the main effect of group were significant [*F*(1, 14) = 5.814, *p* = 0.030, η_*p*_^2^ = 0.293, observed power = 0.612]. Simple-main effects analyses indicated that the control group spent significantly more time in the target quadrant and significantly less time in the other quadrants compared to the T21 rats (target: *p* = 0.031, observed power = 0.608; other: *p* = 0.032, observed power = 0.601).

### c-FOS expression

**FIGURE 4:**
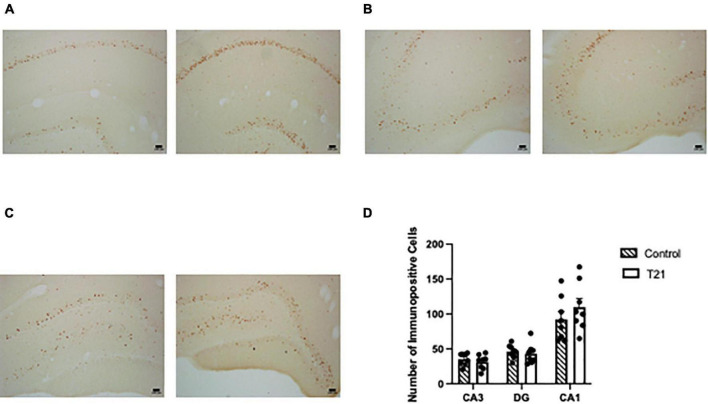
Hippocampal c-FOS expression. The left picture in each figure was taken from a control rat and the right picture in each figure was taken from a T21 rat. For all subregions the pictures were taken from the same control and T21 animal. **(A)** c-FOS expression in CA1. **(B)** c-FOS expression in CA3. **(C)** c-FOS expression in the dentate gyrus. **(D)** Average (± SEM) number of c-FOS+ cells in each subregion of the dorsal hippocampus between groups.

Interrater reliability was very good (K = 0.733, *p* < 0.001). As represented in Figure 4, a MANOVA with CA1, CA3, and DG as the dependent variables failed to find any effect of group [*F*(3, 12) = 0.602, *p* = 0.626].

## Discussion

While there is a spate of evidence demonstrating that circadian rhythms and memory interact, the nature of that relationship is not completely known. The present study was interested in undercovering more about the MWT memory impairment induced by a T21 light dark cycle. Specifically, is a T21 induced memory impairment associated with reduced hippocampal c-FOS expression. Rats were trained on the MWT as in previous paradigms and then received the T21 for 6 days (Devan et al., 2001; Zelinski et al., 2014). T21 exposed rats had impaired MWT retention in the probe trial, but contrary to our hypotheses, hippocampal c-FOS expression was not different from control rats.

In terms of activity rhythms, a lack of a change in period during T21 exposure suggests that the rats were not able to entrain and instead freeran. Freerunning in a short T-cycle has been documented in rodents before (Stephan, 1983; Campuzano, 1998; Cambras et al., 2004, 2007; Vivanco et al., 2010; Casiraghi et al., 2012; Deibel et al., 2020). A delayed phase in the last 6-day block of assessment may indicate that the rats were still not quite entrained to the phase of the finishing light dark cycle. This makes sense as Devan et al. (2001) found that their animals entrained in 17 days. We were replicating the methodology of Zelinski et al. (2014) and they probe tested the animals 13 (19 days after MWT) days after T21 exposure.

In the current study, the finding that memory is impaired when a short T cycle directly follows MWT acquisition, supports others which show that T-cycles or phase advances/delays can impair memory in rodent models (Devan et al., 2001; Craig and McDonald, 2008; Gibson et al., 2010; Loh et al., 2010; Legates et al., 2012; McDonald et al., 2013; Zelinski et al., 2013, 2014; Fernandez et al., 2014; Horsey et al., 2020). Specifically, these data corroborate impairments in the MWT evoked by the T21 using very similar methodology (Devan et al., 2001; Zelinski et al., 2014). It is surprising that 6 days of T21 exposure following learning is sufficient to impair memory 13 days later. Nonetheless, using the same methodology as Zelinski et al. (2014), we have reproduced the memory impairment in MWT retention when learning is immediately followed by 6 days of T21 exposure.

To try to untangle this memory impairment, it is necessary to determine if differences in the amount of neuronal activation are responsible. Ruby et al. (2017) partially addressed this question, by measuring hippocampal c-FOS expression in arrhythmic hamsters that were impaired in a spontaneous alternation task. They found no changes in c-FOS expression, even though this paradigm impairs memory. Their finding required more investigation for several reasons: they only looked in one sub region of the hippocampus and did not assess c-FOS after learning had occurred. In the current study, after memory retention testing in the MWT, there were no differences in c-FOS expression in CA1, CA3, or dentate gyrus.

c-FOS is a transcription factor used as a general indicator of brain activity that can be upregulated in the dentate gyrus, CA1, and CA3 regions of the hippocampus during the MWT (Sagar et al., 1988; Hughes and Dragunow, 1995; Guzowski et al., 2001; Carter et al., 2015; Gallo et al., 2018). Suppressing the expression of c-FOS using central nervous system knockout models or injection of antisense oligonucleotides into the hippocampus can impair memory (Grimm et al., 1997; Guzowski, 2002; Kemp et al., 2013). But these results are contentious and likely depend on factors such as the downregulation method and training regime. Decreases in c-FOS expression are not always associated with cognitive impairments. Antagonism of N-methyl D-aspartate receptors (NMDARs) produced deficits in hippocampal-dependent retention on the MWT task but did not produce a difference in hippocampal expression of c-Fos (Farina and Commins, 2016). Genetic knock out of c-FOS expression solely in the hippocampus also did not impair retention on the hippocampal-dependent MWT and Barnes maze tasks (Zhang et al., 2002). Studies investigating long-term potentiation (LTP) further support the idea that c-FOS might not be a plasticity marker. Anodal direct cranial stimulation produced significant increases in the amplitude and slope of LTP but did not result in increased c-Fos expression following LTP in CA1 and CA3 (Ranieri et al., 2012).

It is important to note that we tested remote memory, which is subject to a process called systems consolidation (Sutherland and Lehmann, 2011; Barry et al., 2016). With the passage of time, memories become less hippocampal dependent and more reliant on neocortical circuits (Sutherland and Lehmann, 2011; Barry et al., 2016). Maybe if we had looked in areas of the neocortex we would have found less c-FOS in the T21 animals. Nonetheless, recall of remote memories does still involve the hippocampus (Sutherland and Lehmann, 2011; Barry et al., 2016). Along these lines, there are other immediate early genes that respond to learning and do so with different time courses (Barry and Commins, 2017). We might have found differing effects on neuronal activation if other immediate early genes were assessed. Related to this, a recent report suggests that the excitation/inhibition state in the circuitry of the dentate gyrus leans more toward inhibition in circadian disrupted animals, without there being any deficit in long term potentiation (McMartin et al., 2021).

One limitation of the present study was that for the significant results we had a middling power of around 60%, meaning that the group sizes were likely on the small side. Future studies investigating this, should use Ns that are bigger than eight. Nonetheless, for MWT, we were still able to detect significant results, which corroborate those found in the literature (Devan et al., 2001; Zelinski et al., 2014).

In the current study, we rule out reduced c-FOS in hippocampal sub-regions as an explanation for memory impairments that are induced by circadian rhythm disruption. When paired with Ruby et al. (2017), it appears that memory impairments induced by photic circadian rhythm manipulations are not a result of attenuated hippocampal c-FOS expression. On the contrary, it does appear that a bias for synaptic inhibition in the dentate gyrus occurs with circadian arrhythmicity (McMartin et al., 2021). But it remains to be seen if this applies to the current data because our animals remained rhythmic during the T21, they were just not entrained to the light dark cycle (Ruby et al., 2008; Fernandez et al., 2014; Shimizu et al., 2016; Kwapis et al., 2018; Hasegawa et al., 2019; Lehr et al., 2021).

## Data availability statement

The original contributions presented in this study are included in the article/Supplementary material, further inquiries can be directed to the corresponding author.

## Ethics statement

All procedures used in the present experiment were conducted in accordance with the Canadian Council of Animal Care Guidelines and were approved by the Institutional Committee on Animal Care at Memorial University.

## Author contributions

SD and SH were involved in the study design and data collection/analysis and wrote the final version of the manuscript. EG was involved in immunohistochemistry scoring. TC and MH-D were involved in data collection. IW was involved in tissue collection and immunohistochemistry. CT oversaw all aspects of the study. All authors contributed to this article and approved the submitted version.

## Supplementary material

Supplementary Figure 1 **(A)** Actograms for control rats. Gray shading represents lights off. Actograms are double plotted. **(B)** Actograms for T21 rats. Gray shading represents lights off. Actograms are double plotted.
