# Supplementary figures and images for: Impaired Morris water task retention following T21 light dark cycle exposure is not due to reduced hippocampal c-FOS expression
Source: Front Behav Neurosci. 2022 Oct 12;16:1025388. doi: 10.3389/fnbeh.2022.1025388 (PMC9596763; doi:10.3389/fnbeh.2022.1025388)

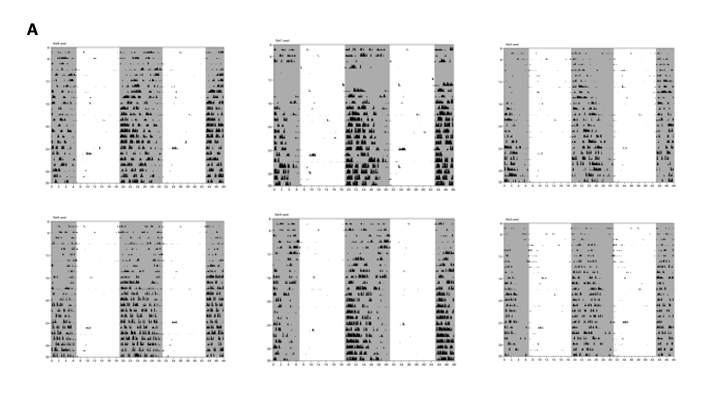

Supplement: Supplementary Figure 1 — (A) Actograms for control rats. Gray shading represents lights off. Actograms are double plotted. (B) Actograms for T21 rats. Gray shading represents lights off. Actograms are double plotted. [file Image_1.TIFF]

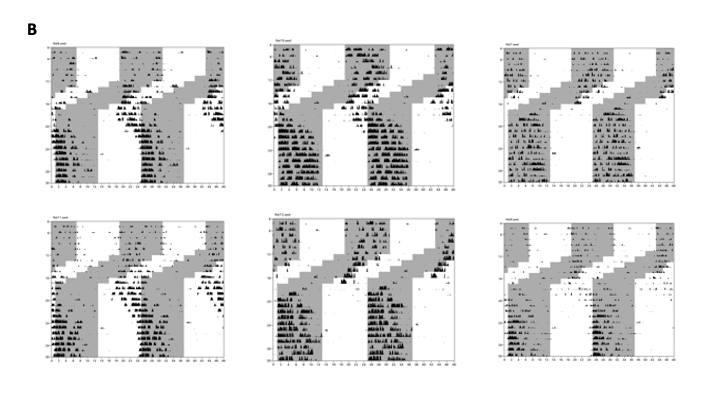

Supplement: Supplementary file 2 [file Image_2.TIFF]
